# Affections of the Heart and Acute Founder

**Published:** 1895-06

**Authors:** M. Bessange

**Affiliations:** Veterinarian at Orleans, France


AFFECTIONS OF THE HEART AND ACUTE FOUNDER IN THE HORSE.—In classical works the etiology of acute founder in the horse includes a mass of varied causes which come for the most part from facts observed by practising veterinarians. There are, however, still numerous cases of founder where the most minute researches cannot reveal any known efficient cause.

I desire to call the attention of my colleagues to a new etiological chapter concerning this disease. In the second year of my practice I observed several successive cases of acute founder in old horses, which worked at light but very regular work, with always identical food, and in which it was impossible to determine any cause which had produced the congestion of the hoofs. A close examination of one of these animals having shown in it a well-developed affection of the heart, my attention was especially called upon this point. Since then I have carefully auscultated the hearts of all foundered animals which I had seen, and the results which I have found authorize me, I believe, to conclude that affections of the heart produce or, at least, favor the production of acute founder, other causes being eliminated.

The following is a summary of eight cases of acute founder concomitant with disease of the heart which I have observed since 1885:

Case I., November, 1886. Large draught gelding, ten years old. Acute founder of both forefeet. Since eight days the horse has worked exclusively at light plowing; has had no change of food for a long time; has had no other known disease within two years. The founder, although severe, has recovered by classical treatment in four days. At my first visit I noticed a slight irregularity of the flank, which I attributed to pain. After the horse recovered the owner told me that the horse had been slightly heavy, but worked well. The pulse was fast, small; auscultation and percussion showed an hypertrophy of the heart and a decided systolic murmur. I diagnosed a chronic endocarditis, without positively finding the point of lesion, which was probably mitral insufficiency. The horse continued work for a year—was then sold.

Case II., February, 1888. Light work gelding, very old. Founder of both forefeet. Regular work at walk; moderately fed; thin, poor condition. Pulse accelerated and irregular. Palpitations evident. Cardiac shock arythmic, diastolic murmur. The founder persisted notwithstanding treatment, and the horse was sent to the knacker. The heart was enormous, surrounded by lesions of chronic pericarditis; the mitral orifice was diminished.

Case III., March, 1890. Mare, used at a trot; about fifteen years old. Moderate, regular work; food regular and light. Feet good. Founder of both forefeet rather severe, requiring treatment for some ten days. Horse in good condition of flesh; flank irregular; dry cough on leaving stable; percussion and auscultation show slight hypertrophy of the heart; doubling of the second sound, which is trembling. No perceptible murmur. During the next year I saw the horse occasionally at my shop. The cardiac symptoms became worse until she could only trot a short distance.

Case IV., September, 1891. Very large draught stallion; works at a walk. A veritable skeleton, about twenty-five years old, and about used. Founder of all four feet, the day after a ten-mile haul. Food could not be a factor, as the horse rarely had oats, finding his food by browsing along the road. The horse made a fair recovery in four days and went back to his collar of misery. He showed all the symptoms of advanced heart-disease: Miserable pulse, hypertrophy of heart, marked systolic murmur. The probable diagnosis was an insufficiency of the aortic valves.

Case V., June, 1892. Gelding, twelve years old. Light work; regular but light diet. Acute founder of all four feet, which answered to treatment in eight days. Mitral insufficiency, slight hypertrophy; irregular respiration, dry cough; thin, emaciated; pulse feeble—fast; jugular pulse; second sound replaced by a murmur. After cure of the founder the horse was only employed at light farm work, and was put under treatment to quiet chronic endocarditis. The founder was radically cured. In two months there was a second attack of founder of the fore-feet, which was slow in recovering and resulted in depressed soles. At this time there was a marked increase in the cardiac symptoms.

Case VI., June, 1893. Horse destroyed for founder, showed marked alterations of semilunar valves of the aorta. White lining showed true intermittence.

Case VII., September, 1893. Stallion, three years old. At eighteen months showed shifting lameness and had been treated by a quack probably for rheumatic synovitis. Horse had only been at light work, but has remained thin with a capricious appetite. Had been attacked with founder in July, 1893, which after treatment by the same quack became chronic. I saw the horse three months later, and found on the two anterior feet enormous cavities and excessively depressed soles. He was hardly able to walk, resting on his side most of the time, until all the bony points were covered with wounds. Respiration was irregular and painful, the pulse small and quick, the shock of the heart arythmic, the second sound double and trembling with a rough murmur at both sounds. The characteristic symptoms I attributed to a lesion of the aortic valve consecutive to the rheumatic synovitis. With careful treatment and proper shoeing the horse was able to walk much better, but was unfit for work which would be remunerative.

Case VIII., August, 1894. Saddle mare, eight years old; has been used as a hunter; now is only used for light work on medium nourishment. Foundered in the two front feet the day after a run of six miles; recovered after five days of treatment. I found a hypertrophy of the heart with doubling of the second sound; no murmur.

That the production of acute founder is favored by the existence of an affection of the heart is not surprising, if one looks into the facts. The congestion of the keratogenous apparatus, whose function is so important and active in the solipeds, can easily be one of the first, after pulmonary congestion, to take place when the course of the blood is interfered with; it is most frequently a mechanical hyperæmia.

The most vascular organs are always those in which congestion takes place most readily, and this last can only be produced by some trouble or other of the circulation: conditions united for explaining logically the production of acute founder (congestion of the podophylleous tissue) by the chronic affection of the heart.

Accordingly as the cardiac alterations take place in one or other of the portions of the heart the congestion will be active or passive, but the results are the same from a clinical point of view. Active congestion produced by an excessive blood pressure can be caused by a hypertrophy of the heart, whatever its cause may be; passive congestion exists always if there is an obstacle to the return of the circulation. Cardiac debility, augmentation of the tension of the venous, is the consequence.

Nearly all authors note that founder may follow the convalescence of severe diseases, such as pneumonia, typhoid fever, anasarca, etc. Hertwig says that he has seen it often after rheumatic affections and especially after acute rheumatism.

Now it is well admitted to-day that these different diseases often leave a chronic endocarditis, with lesions of the different valves. It will be more just to my mind to consider the founder as the result of the cardiac lesion.

The cases that I have given are certainly not numerous enough to be proof, but then they serve as guides to the practitioner who would study more deeply the etiology of acute founder.

Since the time when my attention was called to this point, I have found besides four cases of founder with an obscure etiology in which the most minute examination would not reveal any affection of the heart. In numerous cases of congestion of the foot that I have seen the cause was readily found in the history of severe work, excessive feeding, long idleness in the stable, etc.

In publishing these notes I only desire to submit the interesting question to my colleagues. The future will tell us if the auscultation of the heart of horses affected with founder without appreciable cause will give results identical to those which I have observed.—M. BESSANGE (*Veterinarian at Orleans, France*), *in the Recueil de Médecine Vétérinaire.*

